# Pulmonary Arteriovenous Malformation Causing Systemic Hypoxemia in Early Infancy

**DOI:** 10.1155/2017/2841720

**Published:** 2017-03-08

**Authors:** V. Aggarwal, D. M. Khan, J. F. Rhodes

**Affiliations:** ^1^Department of Pediatric Cardiology, Texas Children's Hospital and Baylor College of Medicine, Houston, TX, USA; ^2^Department of Pediatric Cardiology, Nicklaus Children's Hospital, Miami, FL, USA

## Abstract

Pulmonary arteriovenous malformation (AVM) is not routinely appreciated during the standard echocardiogram to assess for structural abnormalities or pulmonary hypertension. The distal pulmonary AVM is suspected only if an injection of agitated saline is performed and late entry of particles is appreciated in the left heart structures. A large or complex pulmonary AVM can result in significant right-to-left shunting and consequential systemic hypoxemia in the presence or absence of pulmonary hypertension. For direct visualization of the pulmonary AVM, computerized tomography (CT) scan is the procedure of choice. Here, we present two young infants with systemic hypoxemia who underwent standard medical management including mechanical ventilation and one patient was placed on extracorporeal membrane oxygenation (ECMO) before the diagnosis of pulmonary AVM was established. Subsequently, both patients have done well into mid-term follow-up after being treated successfully using transcatheter occlusion techniques in the cardiac catheterization laboratory during early infancy. We aim to emphasize the importance of a high index of suspicion for pulmonary AVM in infants with refractory systemic hypoxemia of unclear etiology.

## 1. Introduction

Systemic hypoxemia in infants is commonly due to pulmonary parenchymal disease, pulmonary hypertension with atrial level right-to-left shunting, structural heart disease, or a severe neurological insult. Once these diagnoses are ruled out the etiology of the hypoxemia often remains unclear. Because systemic hypoxemia is a significant culprit for resulting tissue hypoxia in infants, management strategies include mechanical ventilation, pulmonary vasodilator therapies, and life support strategies such as ECMO. Consequently, if the etiology remains unclear, pulmonary AVM should be considered. Although pulmonary AVM is uncommon, it is a treatable cause of hypoxemia in infants and was first described one hundred and twenty years ago during autopsy studies [1]. It was not until the late 1940s that the first surgical procedures were described [2] and the first transcatheter occlusion of a pulmonary AVM was described in 1978 [3]. Since that time, various devices including Gianturco coils (Cook Medical, Bloomington, IN) [4], detachable balloons (Target Therapeutics, Fremont, OH) [5], Amplatzer® vascular plugs (St. Jude Medical, St. Paul, MN) [6], Amplatzer atrial septal occluder (St. Jude Medical, St. Paul, MN) [7], and Amplatzer patent ductus arteriosus devices (St. Jude Medical, St. Paul, MN) [8] have been used to occlude pulmonary AVMs. We report our experience of two young infants with refractory hypoxemia due to pulmonary AVMs. We emphasize a strong suspicion for the pulmonary AVM in infants with systemic hypoxemia of unclear etiology and a structurally normal heart.

## 2. Case #1

**Figure 1:**
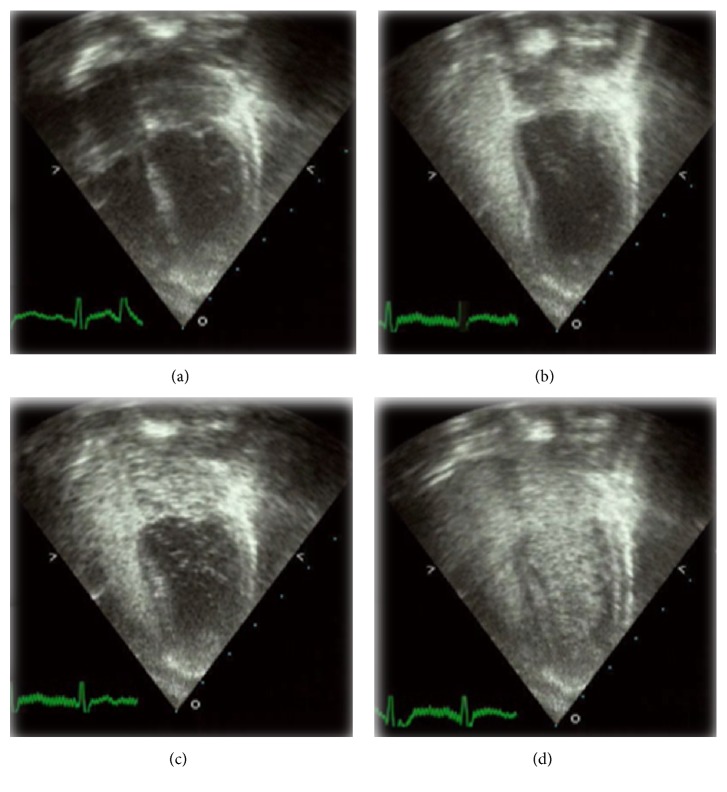
Chest wall echocardiography using agitated saline particles in patient 1 showing echocardiographic still image depicting all four chambers before instillation of agitated saline (a), agitated saline filling the right side of the heart (b), and the left side after a few heart beats (c, d).

**Figure 2:**
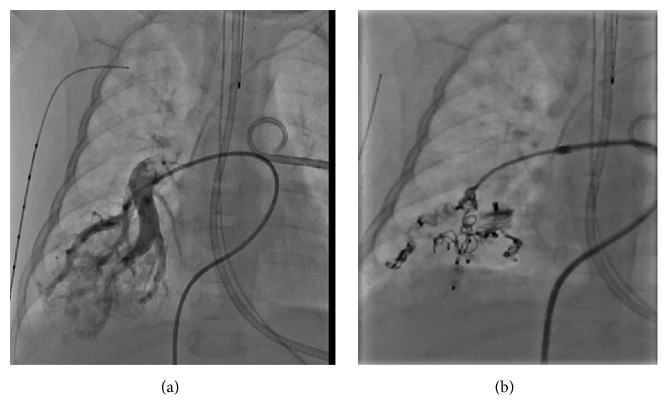
Cardiac catheterization showing the large pulmonary AVM in right lower lobe in patient 1; baseline angiography (a) and after transcatheter occlusion (b).

A four-month-old baby boy that was born full term presented to an outside facility emergency department with parents reporting cough, congestion, and subjective fever for three days. Although he was alert with a normal physical exam and vital signs, his pulse oximetry saturations on room air were between 60 and 70%. His chest X-ray (CXR) showed minimal right lower and middle lobe infiltrates. Echocardiography showed a structurally normal heart and no evidence of pulmonary hypertension. Hypoxemia persisted with pulse oximetry saturations in the mid-70s despite supplemental oxygen therapy using nasal cannula. He was subsequently intubated, ventilated, and started on inhaled Nitric Oxide (iNO) therapy with minimal improvement in his oxygen saturations to the high 70s. Due to persistent hypoxemia, he was transferred to our hospital critical care unit for possible ECMO therapy. Despite high ventilator settings, 100% fractional concentration of inspired oxygen (FiO2), and 80 ppm iNO, his pulse oximetry saturation remained in 70–80%. Partial pressure of oxygen (PaO_2_) obtained via arterial blood gases on this support ranged between 42 and 69 mmHg. Repeat echocardiography confirmed a structurally normal heart, no patent foramen ovale (PFO), and no pulmonary hypertension. Since his low oxygen saturations were out of proportion to the lung disease on CXR, an agitated saline injection with echocardiography of the left heart structures was performed showing delayed (>3 cardiac cycles) entry of particles into the left atrium from the pulmonary veins (Figure 1). Subsequently, a chest CT scan demonstrated the presence of a large (>3 mm) right lower lobe pulmonary AVM with multiple afferent vessels and a single efferent vessel. Family history and evaluation for hereditary thrombophilia telangiectasia (HHT) were both negative. Diverting ECMO therapy temporarily, the patient proceeded for a cardiac catheterization. Systemic heparin was administered to maintain the ACT > 200 seconds. Through a 6 Fr sheath in the right femoral vein, angiography demonstrated a complex single right lower lobe pulmonary AVM with several large arterial afferent vessels (Figure 2). The pulmonary artery that led to the pulmonary AVM was accessed using a 0.035′′ Terumo glide wire (Terumo Medical, Somerset, NJ) through a 6 Fr guide catheter and a telescoped 4 Fr multipurpose catheter (Cook Medical, Bloomington, IN). The AVM was successfully occluded using multiple Magnetic Resonance Imaging (MRI) compatible MReye® coils (Cook Medical, Bloomington, IN) and Amplatzer Vascular Plug–II devices (two 4 mm, two 6 mm, and one 10 mm) (St. Jude Medical, St. Paul, MN). After 5 days of recovery, the infant was discharged home on room air with oxygen saturations of 98–100%. At the last follow-up, after four years, he remains clinically asymptomatic though his room air oxygen saturation had slightly decreased to 92%. The CXR was normal. Chest wall echocardiography demonstrated normal anatomy and no evidence of pulmonary hypertension. Injection of agitated saline particles showed trace to trivial entry of particles into the left heart structures after 3 cardiac cycles. Follow-up chest CT scan is planned at the five-year visit in consideration for possible recurrent pulmonary AVMs.

## 3. Case #2

Baby girl born at 39-week gestational age at an outside facility by uneventful repeat cesarean section developed respiratory distress soon after birth with pulse oximetry saturations of 62% on oxygen therapy. Due to low oxygen saturations refractory to supplemental oxygen, she was intubated and mechanically ventilated. Echocardiography showed bidirectional shunting at the PFO but no other structural heart defects. Flattening of the interventricular septum by echocardiography was suggestive of increased right ventricular systolic pressure. Consequently, iNO and high frequency ventilation were initiated but failed to improve her oxygenation. CXR showed bilateral lung haziness and a left lower lobe airspace opacity. At 3 days of age she was transferred to our hospital critical care unit with a diagnosis of persistent pulmonary hypertension of the newborn and was placed on ECMO support. Pulmonary hypertension was managed with iNO, 100% FiO2, intravenous sildenafil, and inhaled iloprost. By 10 days of age, echocardiography demonstrated normal estimated peak right ventricular pressures. Despite resolution of the pulmonary hypertension, multiple attempts to wean from ECMO failed due to refractory hypoxemia. Other diagnoses such as alveolar capillary dysplasia were also considered and genetic analysis for FOX 1 gene was negative. A trial of surfactant therapy was given but did not result in appropriate clinical improvement. The diagnosis of possible pulmonary AVM was considered and an agitated saline injection during echocardiography of the left heart structures was performed at 16 days of age. Unfortunately, the study was inconclusive due to bidirectional shunting of the agitated saline across the PFO making it difficult to appreciate late entry of the agitated saline into the left heart. Chest CT was deemed high risk due to the ECMO support. Consequently, at 19 days of age, due to a strong clinical suspicion of a pulmonary AVM, the patient underwent cardiac catheterization. Systemic heparin was administered to maintain the ACT > 200 seconds and a 6 Fr sheath was place in the right femoral vein. The left lower pulmonary vein saturation was 70% while all other pulmonary veins were 99%. Pulmonary angiography showed a left lower lobe pulmonary AVM. The pulmonary artery that led to the pulmonary AVM was accessed using a 0.035′′ Terumo glide wire (Terumo Medical, Somerset, NJ) through a 6 Fr guide catheter and a telescoped 4 Fr multipurpose catheter (Cook Medical, Bloomington, IN). The AVM had two afferent arterial vessels which were occluded using Amplatzer Vascular Plug–II devices (4 mm, 8 mm, and two 6 mm devices). The ECMO support was removed in the catheterization laboratory. She was extubated 2 weeks after pulmonary AVM occlusion and discharged home a week later. Follow-up at two years, she remains stable with pulse oximetry saturations of 98% and a normal echocardiogram.

## 4. Discussion

Pulmonary AVMs are abnormal direct connections between a pulmonary artery and a pulmonary vein without an intervening capillary bed. These malformations can exist as a single focal lesion or as multiple lesions and have an incidence of approximately 1 per 100,000 population with a male to female ratio ranging from 1 : 1.5 to 1 : 1.8 [9].

The right-to-left shunting of blood secondary to a pulmonary AVM can result in systemic hypoxemia, paradoxical embolism, stroke, cerebral abscess, or pulmonary hemorrhage. In different series, 13 to 55% of patients with pulmonary AVM were asymptomatic [10–12]. The most common presenting symptom reported in these series is dyspnea on exertion (31% to 67%). In older patients, a meticulous physical examination can detect abnormal physical findings (like cyanosis, clubbing, and pulmonary vascular bruit) in up to 75% of patients [13]. Presentation in neonatal period and infancy is only if the AVM is large enough to cause significant right-to-left shunting of blood. These can however be subtle and nonspecific and diagnosing this condition might be difficult. The uncommon finding of an early infancy pulmonary AVM can result in an unrecognized treatable diagnosis. Both our patients were intubated prior to transfer and diagnosis of pulmonary AVM was not considered initially. Refractory hypoxemia unexplained by CXR findings and with a structurally normal heart by echocardiography should strongly raise the suspicion for an extracardiac right-to-left shunt such as pulmonary AVM.

Contrast echocardiography using agitated saline has nearly 100% sensitivity for screening patients [14, 15] for PAVM. Using contrast echocardiography for screening reduces radiation exposure by limiting CT scan to patients with a positive screening.

The main indication for treatment of pulmonary AVM includes prevention of stroke and cerebral abscess [16], improvement in hypoxemia, exercise tolerance [17], and prevention of lung hemorrhage. Treatment of pulmonary AVM can be surgical lobectomy to minimal invasive transcatheter interventions. Surgical treatment has significant morbidity and mortality and often requires removal of part of the lung that is not involved with the AVM. Surgical therapies refined to pneumonectomy [18] and then lobectomy in 1950 and to local excision in 1959 [19, 20]. Surgery remained the mainstay of treatment until 1978 when Taylor and colleagues [3] reported the first successful percutaneous catheterization and embolization of pulmonary AVM. Currently, embolization is the most commonly used treatment for people with pulmonary AVMs. The advantages of embolization over surgical intervention of pulmonary AVMs are that it is less invasive and easy to repeat. The choice of embolization device depends on the vascular anatomy of the individual. Types of embolization devices include coils and Amplatzer vascular plugs. A recent Cochrane review [21] failed to identify any randomized controlled trial of embolization versus surgery or comparing different embolization devices. The review concludes that the observational studies do suggest a substantial reduction in morbidity and mortality with embolotherapy.

Transcatheter embolization using coils has been reported previously in newborn [21–23]. Stainless Steel (Gianturco) coils have traditionally been used to occlude vascular structures. Stainless steel coils used in the thorax can cause significant artifact on MRI scans of the chest. We have therefore now changed our practice to only using MRI compatible coils. Additional reports [6] reviewed various types of Amplatzer vascular plugs (St. Jude Medical, St. Paul, MN) and described the use for various pathologies including pulmonary AVM. In the literature, the recanalization rate for PAVM is 2.8–15% [24, 25]. The Amplatzer Vascular Plug II (St. Jude Medical, St. Paul, MN) has three discs and six layers of Nitinol mesh, thereby giving it better occlusive properties than Amplatzer Vascular Plug version (St. Jude Medical, St. Paul, MN).

## 5. Conclusions

Pulmonary AVM is a relatively infrequent but an easily treatable cause of systemic hypoxemia during early infancy. Although pulmonary AVMs tend to present in older children and adults in both of our patients the pulmonary AVM caused significant hypoxemia in early infancy. In both of these patients, “normal” echocardiographic studies without appropriate agitated saline injections mislead the treating physicians to pursue a pulmonary parenchymal cause of hypoxemia. We stress a high index of suspicion for pulmonary AVM and a low threshold to perform an agitated saline echocardiography on patients with refractory hypoxemia, a structurally normal heart, and parenchymal lung findings inconsistent with the clinical scenario. With newer transcatheter device technology, pulmonary AVM can be treated in the catheterization laboratory without resorting to surgical resection even in early infancy.

## Abbreviations

AVM: Arteriovenous malformations
CT: Computerized tomography
ECMO: Extracorporeal membrane oxygenation
CXR: Chest X-ray
NO: Inhaled Nitric Oxide
FiO2: Fractional concentration of inspired oxygen
PaO2: Partial pressure of arterial oxygen
PFO: Patent foramen ovale
HHT: Hemorrhagic telangiectasia
MRI: Magnetic Resonance Imaging.
